# Chronic Alterations in Monoaminergic Cells in the Locus Coeruleus in Orexin Neuron-Ablated Narcoleptic Mice

**DOI:** 10.1371/journal.pone.0070012

**Published:** 2013-07-29

**Authors:** Natsuko Tsujino, Tomomi Tsunematsu, Motokazu Uchigashima, Kohtarou Konno, Akihiro Yamanaka, Kazuto Kobayashi, Masahiko Watanabe, Yoshimasa Koyama, Takeshi Sakurai

**Affiliations:** 1 Department of Molecular Neuroscience and Integrative Physiology, Faculty of Medicine, Kanazawa University, Kanazawa, Ishikawa, Japan; 2 International Institute for Integrative Sleep Medicine, University of Tsukuba, Tsukuba, Japan; 3 Research Institute of Environmental Medicine, Nagoya University, Nagoya, Japan; 4 Department of Anatomy and Embryology, Graduate School of Medicine, Hokkaido University, Sapporo, Japan; 5 Department of Molecular Genetics, Fukushima Medical University School of Medicine, Fukushima, Japan; 6 Department of Science and Technology, Fukushima University, Fukushima, Japan; Hospital General Dr. Manuel Gea González, Mexico

## Abstract

Narcolepsy patients often suffer from insomnia in addition to excessive daytime sleepiness. Narcoleptic animals also show behavioral instability characterized by frequent transitions between all vigilance states, exhibiting very short bouts of NREM sleep as well as wakefulness. The instability of wakefulness states in narcolepsy is thought to be due to deficiency of orexins, neuropeptides produced in the lateral hypothalamic neurons, which play a highly important role in maintaining wakefulness. However, the mechanism responsible for sleep instability in this disorder remains to be elucidated. Because firing of orexin neurons ceases during sleep in healthy animals, deficiency of orexins does not explain the abnormality of sleep. We hypothesized that chronic compensatory changes in the neurophysiologica activity of the locus coeruleus (LC) and dorsal raphe (DR) nucleus in response to the progressive loss of endogenous orexin tone underlie the pathological regulation of sleep/wake states. To evaluate this hypothesis, we examined firing patterns of serotonergic (5-HT) neurons and noradrenergic (NA) neurons in the brain stem, two important neuronal populations in the regulation of sleep/wakefulness states. We recorded single-unit activities of 5-HT neurons and NA neurons in the DR nucleus and LC of orexin neuron-ablated narcoleptic mice. We found that while the firing pattern of 5-HT neurons in narcoleptic mice was similar to that in wildtype mice, that of NA neurons was significantly different from that in wildtype mice. In narcoleptic mice, NA neurons showed a higher firing frequency during both wakefulness and NREM sleep as compared with wildtype mice. In vitro patch-clamp study of NA neurons of narcoleptic mice suggested a functional decrease of GABAergic input to these neurons. These alterations might play roles in the sleep abnormality in narcolepsy.

## Introduction

Orexin A and orexin B (hypocretin 1 and hypocretin 2) are hypothalamic neuropeptides implicated in the regulation of sleep/wakefulness states [1], [2].

Orexin neurons diffusely innervate the entire neuroaxis excluding the cerebellum, with particularly dense innervation to monoaminergic/cholinergic nuclei in the brain stem, such as the raphe nuclei, tuberomammillary nucleus (TMN), locus coeruleus (LC), and laterodorsal/pedunculopontine tegmental nuclei (LDT/PPT) [3], [4]. These nuclei are thought to play important roles in sleep/wakefulness regulation.

The role of these monoaminergic/cholinergic neurons in sleep/wakefulness regulation has been clarified by the correlation of their spontaneous firing frequency with the sleep-waking cycle [5]–[10]. Monoaminergic release in several cortical and subcortical sites was also correlated with the sleep-waking cycle [11]–[17].

Several lines of evidence have suggested that orexins maintain wakefulness by regulating these monoaminergic neurons. Firstly, two orexin receptors are differentially expressed in these nuclei [18], [19]. Secondly, electrophysiological studies showed that orexins excite these monoaminergic neurons [20]–[22]. Thirdly, the change in firing rate of orexin neurons showed a similar pattern to that of monoaminegic neurons [23]–[25], with rapid firing during wakefulness and attenuation of firing during sleep.

Selective loss of orexin neurons causes the sleep disorder, narcolepsy, in humans and animals. Although the cardinal symptom of human narcolepsy is severe excessive daytime sleepiness, patients also frequently suffer from insomnia with premature nocturnal awakening. Orexin-deficient mice also show short bouts of both NREM sleep and wakefulness. These mice have more transitions between all states [26]. Abnormality of NREM sleep cannot be simply explained by orexin deficiency, because orexin neurons cease firing during sleep [23]–[25]. Therefore, we hypothesized that chronic compensatory changes in the function of wake-promoting regions might affect NREM sleep in narcolepsy.

In this study, we examined whether the activity of monoaminergic neurons is changed in narcolepsy model mice, in relation to their activities during sleep/wakefulness states. Although 5-HT neurons showed almost normal firing patterns according to behavioral states, NA neurons in the LC showed an altered firing pattern. NA neurons in *orexin/ataxin-3* mice showed higher activity as compared with those in wildtype mice during both wakefulness and NREM sleep, especially in the early epoch of NREM sleep. We also found that the frequencies of sIPSCs and mIPSCs of NA neurons were markedly decreased in *orexin/ataxin-3* mice as compared with wildtype mice, suggesting that the increase in firing rate of NA neurons might be due to alterations in GABAergic input to these cells.

## Results

### Characteristics of Sleep States of Orexin/Ataxin-3 Mice Under Recording Condition

To record single-unit activities of neurons extracellularly in a non-anesthetized mouse, we restrained the mouse in a stereotaxic frame with a plastic plate attached to its skull. To examine the effect of this restrained condition on the sleep/wakefulness cycle, *orexin/ataxin-3* mice and their wildtype littermates were analyzed by simultaneous EEG/EMG recording. We found that the sleep/wakefulness characteristics of *orexin/ataxin-3* mice were comparable to those of wildtype mice under this recording condition in the late light period (from 13∶00 to 18∶00). The percentage of the total of each episode time was similar in both groups (W, 19.6±2.6 vs 24.7±4.3%; NREM sleep, 59.8±2.6 vs 55.4±1.0%; REM sleep, 20.0±2.2 vs 18.7±3.3%; quiet waking (qW), 0.5±0.4 vs 1.1±4.5%, wildtype mice vs *orexin/ataxin-3* mice, t_7_ = 0.74 p = 0.37, t_4_ = 1.68 p = 0.2, t_7_ = 0.30 p = 0.77, t_7_ = 0.97 p = 0.36, respectively). The mean episode durations in *orexin/ataxin-3* mice also did not show a significant difference to those in wildtype mice (W, 16.1±1.4 vs 17.0±1.0 s; NREM sleep, 49.1±6.5 vs 39.8±6.6 s; REM sleep, 91.8±12.5 vs 95.6±10.2 s; qW 5.9±3.4 vs 10.3±2.1 s, wildtype mice vs *orexin/ataxin-3* mice, t_7_ = 0.54 p = 0.60, t_7_ = 0.99 p = 0.35, t_7_ = 0.24 p = 0.81, t_7_ = 1.14 p = 0.29, respectively). There was no significant difference in REM sleep latency between genotypes (65.4±4.5 vs 59.1±4.2 s, wildtype mice vs *orexin/ataxin-3* mice, t_7_ = 1.02 p = 0.34, respectively). Episode number per unit time in *orexin/ataxin-3* mice was not significantly different from that in wildtype mice (n = 10, t_7_ = 1.06 p = 0.32). EEG/EMG recording showed no direct transition from the awake state to REM sleep during the recording time, consistent with a previous report showing that nearly all episodes of cataplexy occurred during the dark period [26], [27].

In our previous report, in a freely moving condition, the percentages of wakefulness and NREM sleep time were almost the same in wildtype and *orexin/ataxin-3* mice. This result was reproduced in a head-restrained condition. Although the total time of REM sleep in the light period was shorter in *orexin/ataxin-3* mice than in wildtype mice in a freely moving condition [27], there was no significant difference between the two genotypes in a head-restrained condition. However, in the head-restrained condition, we observed a relatively longer REM sleep time as compared to that in a freely behaving condition. We also observed a considerably shorter length of NREM sleep (39–49 s). These might possibly be due to the restraint stress. We analyzed the total gathered sleep time data obtained from the mice in which extracellular recordings were successfully obtained. Recordings were lost mainly when mice were in active wakefulness. These conditions made the amount of REM sleep longer in our recording condition than in a normal freely behaving condition.

We also examined whether head-restrained stress equally affected *orexin/ataxin-3* and wildtype mice. *Corticotropin releasing factor* mRNA and serum corticosterone levels in a restrained condition showed no significant difference between these two genotypes (1.80±0.40 vs 1.24±0.08, 237.5±54.9 vs 389.0±45.1, wildtype mice vs *orexin/ataxin-3* mice, t_8_ = 1.34 p = 0.22, t_9_ = 2.07 p = 0.07, respectively). This result suggests that our recording condition evoked similar physiological stress in *orexin/ataxin-3* and wildtype mice.

### Firing of Serotonergic Neurons in Wildtype Mice

**Figure 1:**
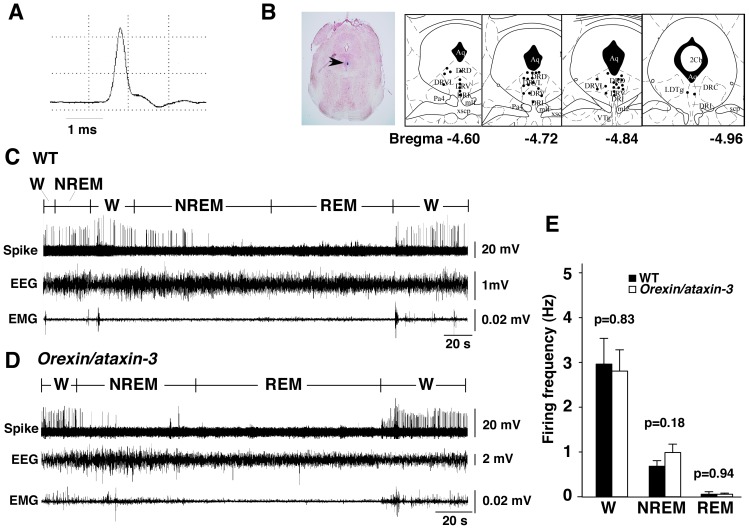
Single unit recordings of 5-HT neurons in dorsal raphe nucleus of wildtype mice and *orexin/ataxin-3* mice in different behavioral states. A, Averaged trace of action potentials of neurons recorded from DR. 5-HT neurons displayed a broad asymmetrical biphasic spike wave form. The first positive component had large amplitude. The descending part often exhibited a shoulder. The negative component decayed slowly and its amplitude was much smaller than that of the positive component. B, Histological verification of recording position. Left panel, Pontamine Sky Blue deposit indicated by an arrowhead is seen in electrode track near the DR. Right panel, Locations of recorded neurons are plotted as black dots on diagrams of serial coronal sections in DR. The number below each section denotes distance (mm) from bregma. C, Representative traces of 5-HT neuron spikes and EEG/EMG recordings in wildtype mice. The firing rate of this neuron increased during W, gradually decreased during NREM sleep, and was inactive during REM sleep. States were judged by EEG and EMG recordings. D, Representative traces of 5-HT neuron spikes and EEG/EMG recordings in *orexin/ataxin-3* mice. Firing patterns across W-NREM sleep and REM sleep-W transitions were not different from those in wildtype mice. E, Firing frequency of 5-HT neurons in wildtype mice (W, NREM, REM: n = 19, 21, 14) and *orexin/ataxin-3* mice (W, NREM, REM: n = 18, 23, 14). Abbreviations: W, waking; DRC, dorsal raphe caudal part; DRD, dorsal raphe dorsal part; DRI, dorsal raphe interfascicular part; DRV, dorsal raphe ventral part; DRVL, dorsal raphe ventrolateral part.

Similarly to in rats, mouse 5-HT neurons were identified by their typical broad spike. In addition, the broad-spike neurons were characterized by their negative component, with a slowly decaying slope being much smaller in amplitude than the positive component and a shoulder on the descending phase of action potentials (Fig. 1A). Histological evaluation validated that the recorded neurons were all located within the area corresponding to the DR (Fig. 1B). Neurons were recorded for 3 to 20 min, and were typically lost during large movements associated with periods of active wakefulness.

Recorded neurons showed changes in spontaneous firing frequency that correlated with the sleep/wakefulness cycle (Fig. 1C). During wakefulness, mean firing frequency was 2.96±0.57 Hz (n = 19). During NREM sleep, these cells showed a decrease in firing frequency to 0.68±0.12 Hz (n = 21). During REM sleep, they nearly ceased firing (0.06±0.05 Hz, n = 14) (Fig. 1E). Coefficient of variation (C.V.) of the spike interval of W, NREM sleep and REM sleep was 0.64±0.05 (n = 19), 0.90±0.13 (n = 21) and 0.26±0.20 (n = 14), respectively.

### Firing of Serotonergic Neurons in Orexin/Ataxin-3 Mice

A total of 24 5-HT neurons in the DR of *orexin/ataxin-3* mice were recorded. The overall firing patterns of 5-HT neurons in *orexin/ataxin-3* mice across the sleep-wakefulness cycle are illustrated in Fig. 1D. Mean firing rates of 5-HT neurons in *orexin/ataxin-3* were 2.80±0.47 Hz (n = 18) during W, 0.98±0.18 Hz (n = 23) during NREM sleep, and 0.05±0.02 Hz (n = 14) during REM sleep (Fig. 1E).

The firing frequency in NREM sleep in *orexin/ataxin-3* mice tended to be higher but the difference did not reach significance (0.98±0.18 and 0.68±0.12 Hz, respectively, t_42_ = 1.37 p = 0.17). Mean firing frequency during W and REM sleep also showed no significant difference. C.V. of the spike interval in W, NREM sleep and REM sleep was 0.59±0.05 (n = 18), 1.12±0.16 (n = 23), and 0.53±0.26 (n = 14). Again, there was no significant difference in this parameter between *orexin/ataxin-3* mice and wildtype mice (t_35_ = 0.62 p = 0.54, t_42_ = 1.06 p = 0.29, t_26_ = 0.81 p = 0.43, respectively). These observations suggest that 5-HT neurons in *orexin/ataxin-3* mice have almost normal firing frequency and normal firing patterns in all sleep/wakefulness states, consistent with a previous report showing that the discharge patterns of serotonergic dorsal raphe cells of narcoleptic dogs across sleep-waking states did not differ from those recorded in normal animals, with tonic discharge in waking, reduced activity in non-REM sleep and cessation of activity in REM sleep [28].

### Firing of Noradrenergic Neurons in Wildtype Mice

**Figure 2:**
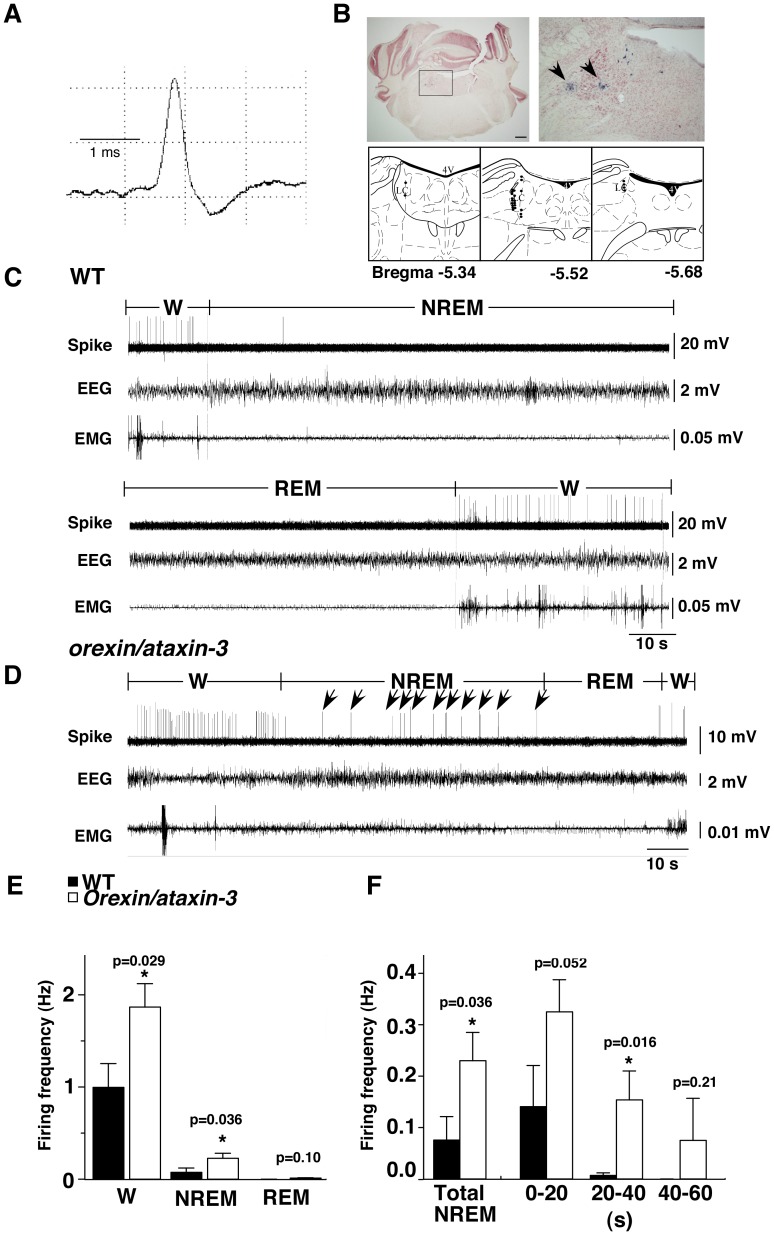
Single unit recordings of NA neurons in LC of wildtype mice and *orexin/ataxin-3* mice in different behavioral states. A, Averaged trace of action potentials of neurons recorded from LC. NA neurons displayed a broad asymmetrical biphasic spike waveform like that of 5-HT neurons in Fig. 1. B, Histological verification of recording position. Upper panels, Typical example of histological appearance. Pontamine Sky Blue deposits indicated by arrowheads are seen in electrode track near LC. Right panel is high power view of rectangular region in left panel. Lower panels, Locations of recorded neurons are plotted as black dots on diagrams of serial coronal sections in LC. C, Representative traces of NA neuron spikes and EEG/EMG recordings in wildtype mice. NA neurons increased firing during wakefulness, almost ceased before entering NREM sleep, and completely ceased firing during REM sleep. D, Representative traces of NA neuron spikes and EEG/EMG recordings in *orexin/ataxin-3* mice. Firing continued after the transition from W to NREM sleep. Arrows indicate the remaining firing during NREM sleep. E, Firing rates of NA LC neurons in wildtype (W, NREM, REM: n = 9, 19, 9) mice and *orexin/ataxin-3* mice (W, NREM, REM: n = 11, 19, 7). Firing rates of LC neurons in *orexin/ataxin-3* mice were significantly higher than those in wildtype during W and NREM sleep. F, Time course of decrease in firing frequency during NREM sleep. NA neurons of *orexin/ataxin-3* mice continued to fire after entry into NREM sleep, especially in the early stage of NREM sleep. Numbers on horizontal axis are time (s) after onset of NREM sleep. At 0 to 40 seconds after the onset of NREM sleep, firing frequency was higher in *orexin/ataxin-3* mice. * p<0.05.

NA neurons were identified mainly by their typical broad action potentials, with a spike shape similar to that of 5-HT neurons. Spontaneous firing frequency during the sleep-waking cycle was used only as a guide (Fig. 2A, C). Histological examination performed by marking the recording position after recording validated that the recording area corresponded to the LC (Fig. 2B). Their mean firing frequency was 1.00±0.26 Hz (n = 9) during W. During NREM sleep, most NA neurons ceased firing 1 to 2 seconds prior to the onset of EEG synchronization (firing frequency was 0.07±0.05 Hz, n = 19). Firing of NA neurons abruptly ceased at the boundaries of W and NREM sleep. This pattern is distinct from the firing pattern of 5-HT neurons, which showed a gradual decrease of firing during NREM sleep (Fig. 2E). This suggests that the firing pattern of NA neurons is more tightly regulated according to vigilance states as compared with 5-HT neurons.

During REM sleep, they were completely silent (firing frequency was 0 Hz, n = 9). C.V. of the spike interval of W, NREM sleep and REM sleep was 0.94±0.10 (n = 9), 0.18±0.08 (n = 19) and 0 (n = 9), respectively.

### Firing of NA Neurons in Orexin/Ataxin-3 Mice

The overall mean firing rates of NA neurons in *orexin/ataxin-3* mice were significantly higher than those in wildtype mice during W (1.87±0.27 Hz, n = 11, t_18_ = 2.37 p = 0.029) and NREM sleep (0.23±0.05 Hz, n = 19, t_36_ = 2.18 p = 0.036) (Fig. 2E). Firing frequency of NA neurons in *orexin/ataxin-3* mice during REM sleep (0.01±0.01 Hz, n = 7) was similar to that in wildtype mice (Fig. 2E). C.V. of the spike interval during W, NREM sleep and REM sleep was 0.70±0.04 (n = 11), 0.75±0.15 (n = 19), and 0.10±0.10 (n = 7), respectively. C.V. during W was significantly smaller than that in wildtype (t_18_ = 2.33 p = 0.04), suggesting that the increase in firing rate of NA neurons during W is due to an increase in persistent synaptic excitatory input and/or a decrease in persistent synaptic inhibitory input to NA neurons rather than periodic phasic excitation of NA neurons. On the contrary, during NREM sleep, C.V. of the spike interval was significantly greater in *orexin/ataxin-3* mice than in wildtype mice (t_36_ = 3.41 p = 0.002), reflecting a relatively more irregular distribution of interspike intervals in *orexin/ataxin-3* neurons during NREM sleep.

During NREM sleep, NA neurons in *orexin/ataxin-3* mice showed significant differences in firing profile in the early time window of NREM sleep as compared with those in wildtype mice. In wildtype mice, NA neurons ceased firing immediately before the onset of EEG synchronization. In contrast, in *orexin/ataxin-3* mice, NA neurons continued to fire after entry into NREM sleep (Fig. 2C, D). This remaining firing lasted at least 60 sec.

We examined the time course of the decrease in firing frequency of NA neurons every 20 seconds from the start of NREM sleep (Fig. 2F). In the first 20-second window, firing frequency of NA neurons in *orexin/ataxin-3* showed a tendency to be higher than that in wildtype (*orexin/ataxin-3*, 0.325 Hz; wildtype, 0.136 Hz, t_33_ = 2.02 p = 0.052). In the next 20-second window, firing frequency in *orexin/ataxin-3* was significantly higher than that in wildtype (*orexin/ataxin-3*, 0.154 Hz; wildtype, 0.006 Hz, t_12.16_ = 2.70 p = 0.019). In the third 20 seconds after NREM sleep onset, although there was no significant difference between genotypes, firing continued during the later portion of NREM sleep in *orexin/ataxin-3* mice (*orexin/ataxin-3*, 0.075 Hz; wildtype, 0 Hz, t_5_ = 1.32 p = 0.36). This occasional firing was not observed in wildtype mice. From these observations, NA neurons in *orexin/ataxin-3* mice seemed to be more excitable during NREM sleep. We also observed periodic firing of NA neurons during REM sleep in *orexin/ataxin-3* mice, further suggesting dysregulation of NA neurons.

Change in the firing frequency of NA neurons is known to be correlated with vigilance level, so we examined whether the vigilance level differed between wildtype and *orexin/ataxin-3* mice in our recording condition. EEG frequency distribution was analyzed by power spectral analysis, Fast Fourier Transformation (FFT), during wakefulness. The percentage of EEG power in the delta, theta, alpha and beta frequency bands showed no significant difference between wildtype and *orexin/ataxin-3* mice (45.2±0.9% vs 42.7±1.0%, t_18_ = 1.85 p = 0.08, 29.9±1.0% vs 32.5±0.9%, t_18_ = 1.99 p = 0.06, 14.2±0.8% vs 13.9±0.5%, t_18_ = 0.41 p = 0.69, 10.7±0.7% vs 10.8±0.3%, t_18_ = 0.16 p = 0.87 for delta, theta, alpha and beta frequency, respectively). This result suggests that the vigilance level during wakefulness was not significantly different between wildtype and *orexin/ataxin-3* mice in this recording condition.

We next examined whether the firing pattern of NA neurons at the transition from sleep to wakefulness differed between the two genotypes. Almost all NA neurons of both genotypes started firing before the onset of EEG activation. This result is in good agreement with a previous report [29]. Of a total of 15 neurons in wildtype mice, 13 NA neurons discharged prior to, 2 at the same time as, and 0 after the onset of W. In *orexin/ataxin-3* mice, firing showed a similar pattern; 20 of 21 discharged prior to and 1 at the same time as the onset of W, and no neuron discharged after the onset of W.

These results indicate that the activity profile of LC neurons during the transition from NREM sleep to W in *orexin/ataxin-3* mice was similar to that in wildtype mice.

### Spontaneous IPSC Frequency of NA Neurons is Decreased in Narcoleptic Mice

Since we found an abnormality in the firing pattern of NA neurons in *orexin/ataxin-3* mice during wakefulness and NREM sleep, we hypothesized that alterations of inhibitory and/or excitatory synaptic input of these neurons occurred in *orexin/ataxin-3* mice. To evaluate this hypothesis, we examined the frequency of sIPSCs and sEPSCs in these neurons. For electrophysiological recording, we used transgenic mice expressing GFP exclusively in tyrosine hydroxylase (TH)-producing neurons (*Th-gfp* mice). *Th-gfp;orexin/ataxin-3* double transgenic (*Th-gfp;orexin/ataxin-3*) mice were used as a narcolepsy model.

First, we examined basic electrophysiological characteristics of NA neurons of *orexin/ataxin-3* mice. Membrane potential, firing frequency and membrane capacitance of noradrenergic neurons in wildtype mice and *orexin/ataxin-3* mice were 44.9±1.3 mV vs 44.6±1.2 mV, t_25_ = 0.25 p = 0.81, 5.14±0.51 Hz vs 5.34±0.38 Hz, t_23_ = 0.23 p = 0.78, and 62.3±5.8 pF vs 60.6±4pF, t_12_ = 0.21 p = 0.84, respectively. These results suggest that the basic characteristics of NA neurons are not altered in *orexin/ataxin-3* mice.

**Figure 3:**
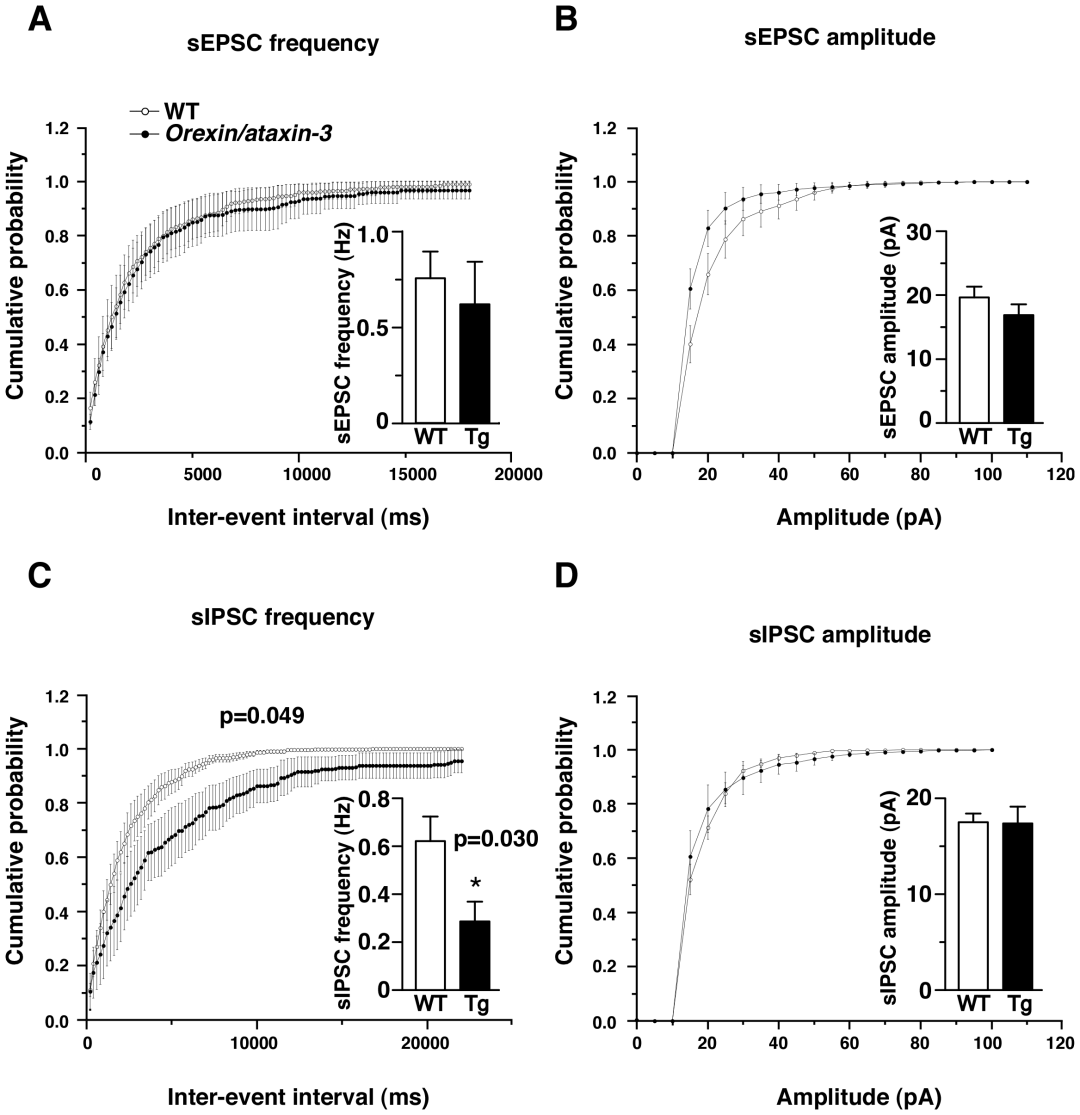
Spontaneous EPSCs and IPSCs in wildtype and *orexin/ataxin-3* mice. We crossed these mice with *Th-GFP* mice for identification of NA neurons. sEPSCs and sIPSCs were recorded from NA neurons in the LC under whole cell patch clamp recording at a holding potential of −60 mV. NA neurons were identified by green fluorescence. A. Cumulative probability plots of sEPSC inter-event intervals of wildtype indicated by open circles and *orexin/ataxin-3* indicated by closed circles (n = 7, 200 ms bin). Inset in A shows mean sEPSC frequency of wildtype mice (WT) and *orexin/ataxin-3* mice (Tg). B. Cumulative probability plots of sEPSC amplitude. Inset shows mean sEPSC amplitude of wildtype mice (WT) and *orexin/ataxin-3* mice (Tg). C. Cumulative probability plots of sIPSC inter-event intervals (n = 6–7), showing decrease in sIPSC frequency in *orexin/ataxin-3* mice. D. Cumulative probability plots of sIPSC amplitude. * p<0.05.

Next, we examined spontaneous EPSCs and IPSCs (sEPSCs and sIPSCs) in NA neurons. There was no significant difference in frequency of sEPSCs between wildtype mice and *orexin/ataxin-3* mice (wildtype, 0.76±0.14Hz, n = 7; *orexin/ataxin-3*, 0.62±0.22Hz, n = 7, t_12_ = 0.52 p = 0.61) (Fig. 3A). Amplitude of sEPSCs showed no significant difference (Fig. 3B) (wildtype: 19.6±1.7 pA, n = 7, *orexin/ataxin-3*∶16.9±1.7 pA, n = 7, t_12_ = 1.18 p = 0.26).

However, the frequency of spontaneous sIPSCs was significantly lower in *orexin/ataxin-3* mice (0.29±0.08 Hz, n = 6) than in wildtype mice (0.62±0.10 Hz, n = 7, F_1.11_ = 4.47 p = 0.05) (Fig. 3C). Amplitude of sIPSCs showed no significant difference (wildtype: 17.5±0.9 pA, n = 7, *orexin/ataxin−3*∶17.4±1.7 pA, n = 6, t_11_ = 0.06, p = 0.95) (Fig. 3D).

**Figure 4:**
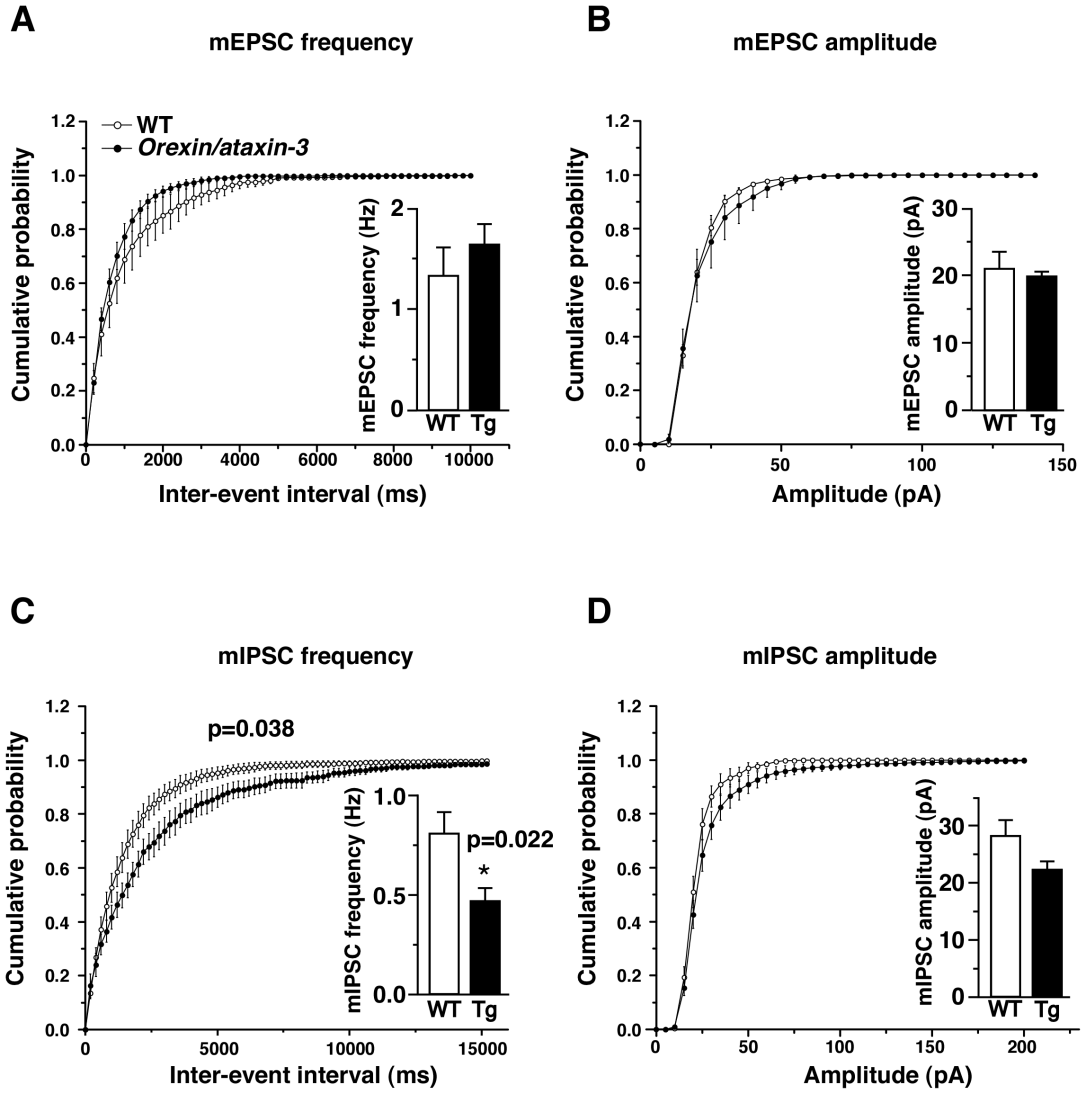
Miniature EPSCs and IPSCs in wildtype and *orexin/ataxin-3* mice. The frequency of mIPSCs was decreased in LC neurons of *orexin/ataxin-3* mice. A. mEPSCs were recorded by whole cell patch clamp at a holding potential of −60 mV. Cumulative probability plots of mEPSC inter-event intervals of wildtype indicated by open circles and *orexin/ataxin-3* indicated by closed circles (n = 6–8, 200 ms bin). Inset in A shows mean mEPSC frequency of wildtype mice (WT) and *orexin/ataxin-3* mice (Tg). B. Cumulative probability plots of mEPSC amplitude. Inset shows mean mEPSC amplitude of wildtype mice (WT) and *orexin/ataxin-3* mice (Tg). C. Cumulative probability plots of mIPSC inter-event intervals (n = 8–10), showing decrease in mIPSC frequency in *orexin/ataxin-3* mice. D. Cumulative probability plots of mIPSC amplitude. * p<0.05.

Next, we examined miniature EPSCs (mEPSCs) and miniature IPSCs (mIPSCs) of NA neurons in *orexin/ataxin-3* mice. There was no significant difference in frequency of mEPSCs between *orexin/ataxin-3* mice and wildtype mice (wildtype: 1.33±0.28 Hz, n = 6, *orexin/ataxin−3*∶1.64±0.21 Hz, n = 8, t_12_ = 0.91 p = 0.38) (Fig. 4A). Amplitude of mEPSCs also showed no significant difference (Fig. 4B) (wildtype: 21.0±2.5 pA, n = 6, *orexin/ataxin−3*∶19.9±0.7 pA, n = 8, t_12_ = 0.90 p = 0.63).

However, frequency of mIPSCs was significantly lower in *orexin/ataxin-3* mice (0.46±0.07 Hz, n = 8) than in wildtype mice (0.80±0.10 Hz, n = 10, F_1.16_ = 5.07 p = 0.04) (Fig. 4 C). Amplitude of mIPSCs showed no significant difference (wildtype: 28.1±2.8 pA, n = 10, *orexin/ataxin−3*∶22.2±1.5 pA, n = 8, t_16_ = 1.71, p = 0.11) (Fig. 4D).

### Overall Density of Inhibitory Synapses in Orexin/Ataxin-3 Mice is not Altered

**Figure 5:**
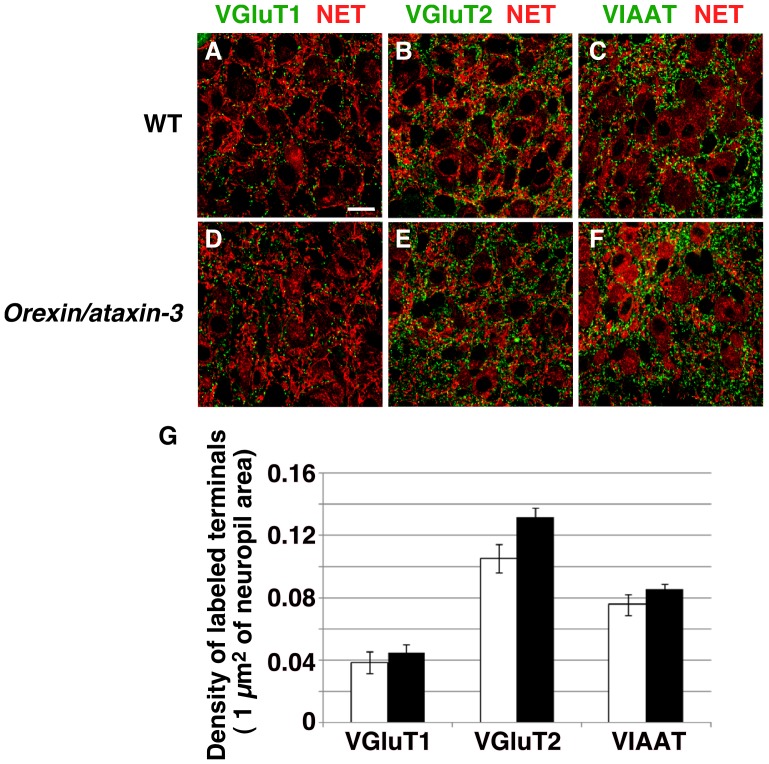
Expressions of norepinephrine transporter (red) and GABAergic/glutamatergic terminal markers (green) in LC of wildtype mice (A–C) and *orexin/ataxin-3* (D–F) mice. A, D, VGluT1; B, E, VGluT2; C, F, VIAAT. Each of the neurochemically-identified terminals is distributed in the neuropil around NET-stained perikarya and dendritic processes of LC neurons. G. Density of VGluT1- (left), VGluT2- (middle), and VIAAT-labeled (right) terminals per 1 µm^2^ of neuropil area. The total measured area was 55,245 and 50,018 µm^2^ for VGluT1, 43,658 and 49,415 µm^2^ for VGluT2, and 67,799 and 54,575 µm^2^ for VIAAT in wildtype and *orexin/ataxin-3* mice, respectively. Scale bar, 20 µm.

To decipher the neuronal mechanism by which IPSCs are decreased in the LC of *orexin/ataxin-3* mice, we examined GABAergic and glutamatergic synapses in the LC by double immunofluorescent staining for norepinephrine transporter (NET) (red in Fig. 5A–F) to define the region containing NA neurons and for neurochemical terminal markers vesicular glutamate transporter (VGluT)1, VGluT2, and vesicular inhibitory amino-acid transporter (VIAAT) (green). In both *orexin/ataxin-3* mice and wildtype littermates, VGluT1-, VGluT2-, and VIAAT-positive terminals were present in the neuropil, and distributed around NET-stained somata and dendrites of LC neurons.

We did not detect any obvious change in density of excitatory and inhibitory terminals. This was confirmed by no significant difference in the density of VGluT1-, VGluT2, and VIAAT-immunostained terminals in the neuropil (n = 4–5 images from each mouse, p>0.05, Fig. 5G).

These results suggest that the increase in firing frequency during W and NREM sleep in *orexin/ataxin-3* mice might be at least partly due to decreased activity of inhibitory neurons projecting to NA neurons, rather than a decrease in number of GABAergic synaptic innervations to NA neurons.

## Discussion

Since orexins are thought to play an important role in regulation of monoaminergic neurons, it is reasonable to hypothesize that orexin neuronal loss leads to alterations in functions of monoaminergic neurons. Previous studies reported that, in the narcoleptic dog, noradrenergic cells of the locus coeruleus ceased discharge during cataplexy, while dorsal raphe REM sleep-off neurons did not cease discharge during cataplexy [28], [30]. However, no report has thus far examined abnormality of firing patterns of these neurons during the transitions of behavioral states.

In this study, we recorded single-unit activities of 5-HT neurons and NA neurons in the DR and LC of wildtype and narcoleptic mice. These nuclei receive major orexinergic innervations and are known to be involved in sleep/wakefulness regulation. We found that the firing pattern of NA neurons was markedly altered in narcoleptic mice, and this change might be due to functional change of GABAergic input to these cells.

Our previous studies showed that *orexin/ataxin-3* mice had reduced mean wakefulness duration and increased number of episodes of wakefulness in a freely moving condition during the dark period [27]. Orexin knockout mice and *orexin/ataxin-3* mice also showed fragmentation of NREM sleep [26], which is consistent with the nocturnal awakenings often seen human narcolepsy. This abnormality of NREM sleep, characterized by difficulty in maintaining consolidated NREM sleep, cannot be simply explained by orexin deficiency, because orexin neuronal activity ceases during NREM sleep [25]. Therefore, we hypothesized that chronic alteration of orexin receptor-expressing neurons, due to compensatory changes of these cells, might be responsible for the instability of NREM sleep in *orexin/ataxin-3* mice. To evaluate this possibility, we performed in vivo extracellular recording of NA neurons and 5-HT neurons in these mice.

As compared to rats, 5-HT neurons in the DR of mice demonstrated lower firing frequency during wakefulness, while the firing frequency of these neurons during NREM sleep and REM sleep exhibited a similar pattern to that in rats [22], [31]. Spike form was nearly identical to that in rats [31], [32]. 5-HT neurons of both wildtype mice and *orexin/ataxin-3* mice fired rapidly during W, decreased during NREM sleep, and ceased during REM sleep in a similar manner (Fig. 1C, D). There was no significant difference in the firing frequency of 5-HT neurons between wildtype mice and *orexin/ataxin-3* mice (Fig. 1E). These results suggest that chronic deficiency of orexin neurons does not have large impact on the firing pattern of 5-HT neurons in DR during sleep/wake states in this experimental condition, although during NREM sleep, firing frequency tended to be increased in *orexin/ataxin-3* mice (Fig. 1E).

On the other hand, NA neurons of *orexin/ataxin-3* mice showed significantly higher firing frequency than that in wildtype mice during W and NREM sleep (Fig. 2E). In wildtype mice, unlike 5-HT neurons in the DR, NA neurons in the LC almost completely ceased firing before the onset of cortical EEG synchronization (Fig. 2C). This firing pattern is in good agreement with a previous report [29]. However, NA neurons in *orexin/ataxin-3* mice showed residual firing after the transition to NREM sleep (Fig. 2F). Since noradrenergic tone plays an important role in arousal, the increase in firing of NA neurons in the LC during NREM sleep might contribute to the instability of NREM sleep in narcolepsy and might be related to the frequent nocturnal awakenings in narcoleptic patients.

Moreover, the firing rate of NA neurons during W was also increased in *orexin/ataxin-3* mice (Fig. 2E). These observations suggest that an increased firing rate of NA neurons, causing imbalance between NA neurons and 5-HT neurons, might possibly be related to behavioral arrests seen in *orexin/ataxin-3* mice [27]. Conversely, the change in firing pattern of NA neurons at the transition from sleep to wakefulness in *orexin/ataxin-3* mice was comparable to that in wildtype mice. These neurons increase firing before the end of NREM sleep and thereby herald the return of the waking state by several seconds. This suggests that the increase in firing rate of NA neurons upon wakening is not dependent on the orexin system.

In addition, we found that the frequencies of sIPSCs and mIPSCs of NA neurons are significantly decreased in *orexin/ataxin-3* mice (Figs. 3, 4). However, we could not detect a histological change of synapses in the LC (Fig. 5). Therefore, functional, rather than structural changes of synapses might occur in *orexin/ataxin-3* mice.

The inactivation of NA neurons during sleep is thought to be due to tonic GABAergic inhibition. Moreover, during W, NA neurons are under GABAergic inhibitory tone [33]. It was recently proposed that their inactivation during REM sleep is due to tonic GABAergic inhibition arising from neurons located in the dorsal paragigantocellular reticular nucleus (DPGi) [34] and ventrolateral PAG (vlPAG) [35]. This GABAergic inhibition is thought to increase progressively during NREM sleep and REM sleep. Our present study suggests this GABAergic input to NA neurons might be altered in narcoleptic mice. Alternatively, it is possible that input from local GABAergic neurons is decreased, because there are OX1R- and OX2R-positive GABAergic interneurons in and around the LC [18].

The molecular mechanisms underlying the changes in GABAergic input to orexin neurons should be addressed in future studies. The reduced GABAergic input might result from compensatory changes of GABAergic input, with reduced net excitation of NA neurons due to loss of orexin neurons. These compensatory processes might explain why narcoleptics show an unstable NREM sleep state as well as an unstable wakefulness state.

## Materials and Methods

### Animals

All experimental procedures involving animals were approved by the Kanazawa University Animal Care and Use Committee and were conducted in accordance with NIH guidelines. All efforts were made to minimize animal suffering and discomfort and to reduce the number of animals used. Extracellular recordings were performed on male *orexin/ataxin-3* hemizygous transgenic mice [27] and their wildtype littermates as control. Mice with both genotypes (30–35 g body weight, 8–12 months old) were crossed to (with) C57BL/6J mice at least seven times. Tyrosine hydroxylase-GFP (*Th-gfp)* mice [36], in which green fluorescent protein in the tyrosine gene is specifically expressed in tyrosine hydoroxylase-expressing cells, were used in patch clamp recordings. Patch-clamp recordings were performed on *orexin/ataxin-3* and *Th-gfp* hemizygous double transgenic (*Th-gfp;orexin/ataxin-3*) mice and *Th-gfp* hemizygous transgenic mice as controls. Mice were maintained under a strict 12 hour light:dark cycle (light on at 8∶45 a.m., off at 8∶45 p.m.) in a temperature- (22°C) and humidity-controlled room and fed ad libitum.

### Drugs

6-Cyano-7-nitroquinoxaline-2,3-dione (CNQX), GABA, glutamate, DL-2-amino-5-phosphono-pentanoic acid (AP-5) (Sigma, St. Louis, MO, USA), and tetrodotoxin (TTX) (Wako, Osaka, Japan) were dissolved in physiological solution and applied by bath application. Picrotoxin was dissolved in ethanol and applied by bath application.

### Extracellular Recording

Neuronal recording was performed on mice in an unanesthetized, head-restrained condition. Mice underwent operation under pentobarbital anesthesia (50 mg/kg i.p.) according to a procedure described previously [37], [38]. In brief, electrodes for recording electroencephalographic (EEG) and neck electromyographic (EMG) activity were implanted, and a U-shaped plastic plate was attached to the skull using dental acrylic cement so that the mouse’s head was fixed to the stereotaxic frame. After a 7-day recovery period, they were placed in a plastic box, and the U-shaped plate on their head was fixed to a stereotaxic frame. After habituation for at least five days, recording was performed from 13∶00 to 18∶00.

Single neuronal activity was recorded extracellularly through a glass pipette microelectrode filled with 0.5 M sodium acetate solution containing 2% Pontamine Sky Blue. The neuronal activity was amplified, and filtered with a high-pass cut-off frequency of 10 Hz and a low-pass cut-off frequency of 10 kHz. The activity was digitized at a sampling rate of 10 kHz with a CED 1410 data processor (Cambridge Electronic Design). 5-HT neurons in the dorsal raphe (DR) and NA neurons in the LC were discriminated from others by their longer-duration action potentials (time from onset of positive component to bottom of negative component >0.9 ms), a shoulder on the falling phase (Figs. 1A, 2A), and the firing pattern during sleep-wakefulness cycles: tonic firing during wakefulness, decrease in frequency during slow wave sleep, and complete cessation of firing during REM sleep [5], [29].

To mark the locations of neurons from which we recorded, Pontamine Sky Blue was injected from the recording electrode by passing a negative current (10–15 µA for 6 min) to one or two recording sites in each electrode track. After the experiment, the animals were deeply anesthetized with pentobarbital and perfused through the left cardiac ventricle with 20 ml PBS followed by 20 ml of 4% paraformaldehyde in 0.1 M phosphate buffer. The brain was then removed, postfixed in the same fixative for 24 hr, and sectioned in the coronal plane at a thickness of 40 µm. The sections were then stained with Neutral Red. Nicotinamide adenine diphosphate (NADPH)-diaphorase histochemical staining was used to visualize especially the serotonergic neurons in the DR. Brains and blood samples from some mice were subjected to measurements of CRH and corticosterone levels after the recording experiments.

### EEG and EMG Recording

Male mice were anesthetized and chronically implanted for continuous monitoring of EEG/EMG as described previously (Chemelli et al., 1999). Animals were housed with a 12 hr light/dark cycle and allowed to habituate to recording conditions for 1 week. Four male transgenic mice and three matched wild-type littermates were subjected to recording concurrently. EEG/EMG signals were amplified and filtered (EEG: 0.3–100 Hz, EMG: 30–300 Hz) before being digitized at a sampling rate of 250 Hz, and displayed on a polygraph system. EEG/EMG records were visually scored into 4-s epochs of wakefulness, REM, and non-REM sleep according to standard criteria of rodent sleep [39]. Vigilance states of mice were divided into wakefulness (W), non-rapid eye movement sleep (NREM sleep) and rapid eye movement sleep (REM sleep). Quiet wakefulness (qW) was defined as the period of wakefulness with no or very low locomotor activity and whisker movement, which is often observed before entering NREM sleep.

### Brain Slice Preparation

*Th-gfp;orexin/ataxin-3* mice and *Th-gfp* hemizygous transgenic mice (6–8 weeks old) were anesthetized with isoflurane (Abbott, Osaka, Japan). The mice were decapitated under deep anesthesia. Brains were isolated in ice-cold cutting solution consisting of (mM): 280 sucrose, 2 KCl, 10 HEPES, 0.5 CaCl_2_, 10 MgCl_2_, 10 glucose, pH 7.4, bubbled with 100% O_2_. Brains were cut coronally into 300-µm slices with a microtome (VTA-1000S, Leica, Germany). Slices containing the LC were transferred for at least 1 h into an incubation chamber at room temperature (RT; 24–26°C) filled with extracellular solution containing (mM): 135 NaCl, 5 KCl, 1 CaCl_2_, 1 MgCl_2_, 10 HEPES, 10 glucose, pH 7.4.

### Patch Clamp Recording

*Th-gfp* mice *and Th-gfp;orexin/ataxin-3* double transgenic mice were used for whole cell patch clamp recordings. The slices were transferred to a recording chamber (RC-27L, Warner Instrument Corp., CT, USA) at 32°C on a fluorescence microscope stage (BX51WI, Olympus, Tokyo, Japan). EGFP is expressed in tyrosine hydroxylase neurons in the *Th-gfp* mouse brain, and neurons that showed GFP fluorescence were used for patch clamp recordings [36], [40]. The fluorescence microscope was equipped with an infrared camera (C-3077, Hamamatsu Photonics, Hamamatsu, Japan) for infrared differential interference contrast (IR-DIC) imaging and a CCD camera (JK-TU53H, Olympus) for fluorescent imaging. Recordings were carried out with an Axopatch 200B amplifier (Axon Instruments, Foster City, CA) using a borosilicate pipette (GC150-10, Harvard Apparatus, Holliston, MA) prepared using a micropipette puller (P-97, Sutter Instruments, Pangbourne, UK) and filled with intracellular solution consisting of (mM): 125 K-gluconate, 5 KCl, 1 MgCl_2_, 10 HEPES, 1.1 EGTA-Na_3_, 5 MgATP, 0.5 Na_2_GTP, pH7.3 with KOH. Osmolarity of the solution was checked with a vapor pressure osmometer (model 5520, Wescor, Logan, UT). The osmolarity of the internal and external solutions was 280–290 and 320–330 mOsm/l, respectively. The liquid junction potential of the patch pipette and perfused extracellular solution was estimated to be −16.2 mV and was applied to the data. The recording pipette was under positive pressure while it was advanced toward an individual cell in the slice. A tight seal of 0.5–1.0 GΩ was made by applying negative pressure. The membrane patch was then ruptured by suction. The series resistance during recording was 10–25 MΩ and was compensated. The reference electrode was an Ag-AgCl pellet immersed in the bath solution. During recordings, cells were superfused with extracellular solution at a rate of 1.0–2.0 ml/min using a peristaltic pump (K.T. Lab, Japan).

Spontaneous excitatory postsynaptic currents (sEPSCs), spontaneous inhibitory postsynaptic currents (sIPSCs), miniature excitatory postsynaptic currents (mEPSCs) and miniature inhibitory postsynaptic currents (mIPSCs) were recorded in NA neurons under whole cell voltage clamp mode at a holding potential of −60 mV. sEPSCs were recorded using KCl-based pipette solution consisting of (mM): 145 KCl, 1 MgCl_2_, 10 HEPES, 1.1 EGTA-Na_3_, 2 MgATP, 0.5 Na_2_GTP, pH7.3 with KOH and the sodium channel blocker QX-314 (1 mM) to inhibit action potentials in the recording neuron and in the presence of picrotoxin (100 µM) in the extracellular solution. sIPSCs were recorded using KCl-based pipette solution containing QX-314 (1 mM) in the presence of AP-5 (50 µM) and CNQX (20 µM) in the extracellular solution. mEPSCs were recorded using KCl-based pipette solution in the presence of picrotoxin (100 µM) and tetrodotoxin (1 µM) in physiological solution consisting of (mM): 125 NaCl, 2.5 KCl, 1.25 NaH_2_PO_4_, 26 NaHCO_3_, 2 CaCl_2_, 1 MgSO_4_, 11 glucose, bubbled with 95% O_2_ and 5% CO_2_. mIPSCs were recorded using KCl-based pipette solution in the presence of AP-5 (50 µM), CNQX (20 µM) and tetrodotoxin (1 µM) in physiological solution. The frequencies of sEPSCs, sIPSCs, mEPSCs and mIPSCs were measured using Minianalysis software; only those events with amplitude >10 pA were used. Frequency and amplitude were presented as the mean of 200 sec duration.

### Immunohistochemistry

Mice were fixed transcardially with 4% paraformaldehyde in 0.1 M sodium phosphate buffer (pH 7.2, PB) followed by 4 hr postfixation of excised brains at 4°C. Paraffin sections through the LC were incubated with 10% normal donkey serum for 20 min, a mixture of primary antibodies overnight (1 µg/ml for each), and a mixture of Alexa Fluor 488-conjugated (Invitrogen, Carlsbad, CA) and Cy3-labeled (Jackson ImmunoResearch, West Grove, PA) secondary antibodies (1∶200) for 2 hr. As primary antibodies, we used guinea pig or goat anti-plasmalemmal epinephrine transporter (NET) [41], rabbit anti-type 1 and 2 vesicular glutamate transporters (VGluT1 and VGluT2) [42], and rabbit anti-vesicular inhibitory amino acid transporter (VIAAT) [43] antibodies. Images were taken with a laser scanning microscope (FV1000, Olympus, Tokyo, Japan) equipped with a HeNe/Ar laser system. To avoid bleed-through into adjacent detection channels, Alexa 488 and Cy3 were excited sequentially using the 488 nm and 543 nm excitation laser lines, respectively, and emissions were collected using a spectral detection system configured with a galvanometer diffraction grating in combination with a variable slit for high-resolution wavelength separation. Images were acquired using a PlanApoN (60×/1.42, oil immersion) objective lens (Olympus) with an appropriated pinhole to obtain one Airy unit for 496–508 nm emission wavelengths (optical section thickness, 1.0 µm). Images were captured with confocal software (FV10-ASW, ver.1.7, Olympus), and digitized at 12 bit resolution into an array of 640×640 pixels (pixel size, 0.1 µm). To compare the density of immunostained nerve terminals in the neuropil of the LC, we measured the number of immunostained terminals and the area of neuropil, excluding neuronal somata and capillaries, using MetaMorph software (Molecular Devices, Downingtown, PA). Genotypic differences in terminal density were statistically evaluated by Student’s *t*-test.

### Tissue and Blood Collection and Serum Analysis

To determine the effect of a restrained condition on serum corticosterone level, blood from wild type and *orexin/ataxin-3* mice was collected under deep anesthesia. About 400–500 µl blood was collected from the heart. Blood was incubated at 37°C for 30 min, then at 4°C for 12 h followed by centrifugation at 4°C (1200 g, 30 min) to collect serum. The brain was carefully removed and the hypothalamus collected. Samples of the brain and serum were stored at −80°C until measurement. The level of serum corticosterone was determined by a solid-phase radioimmunoassay.

### CRF mRNA

Total RNA was isolated from the hypothalamus of restrained mice using a RNeasy lipid tissue minikit (Qiagen) according to the manufacturer’s instructions. cDNA was generated from 500 ng of total RNA using Superscript III First-Strand Synthesis Supermix for qRT-PCR (Invitrogen). qPCRs was performed in a LightCycler® 480 II (Roche Applied Science) with an appropriate Universal Probe (Roche). mRNA expression of the CRF and β-actin genes was measured using LightCycler® 480 software version 1.5.0 (Roche Applied Science). mRNA quantification of each target gene under each condition was normalized to β-actin.

### Statistical Analysis

Data were analyzed by unpaired Student’s *t-*test using the Stat View 5.0 software package for Macintosh (Abacus Concepts, Berkeley, CA, USA). Data of cumulative probability plots of EPSC and IPSC were analyzed by repeated measures ANOVA. Probability (*p*)-values less than 0.05 were considered statistically significant.
